# Supplementary material for: A single immunization with spike-functionalized ferritin vaccines elicits neutralizing antibody responses against SARS-CoV-2 in mice
Source: bioRxiv. 2020 Aug 28:2020.08.28.272518. Preprint. [Version 1] doi: 10.1101/2020.08.28.272518 (PMC7457616; doi:10.1101/2020.08.28.272518)
Supplement: 1 [file NIHPP2020.08.28.272518-supplement-1.pdf]

bioRxiv preprint doi: https://doi.org/10.1101/2020.08.28.272518. this version posted August 28, 2020. The copyright holder for this preprint (which was not certified by peer review) is the author/funder. It is made available under a CC-BY 4.0 International license.

Supplemental Materials

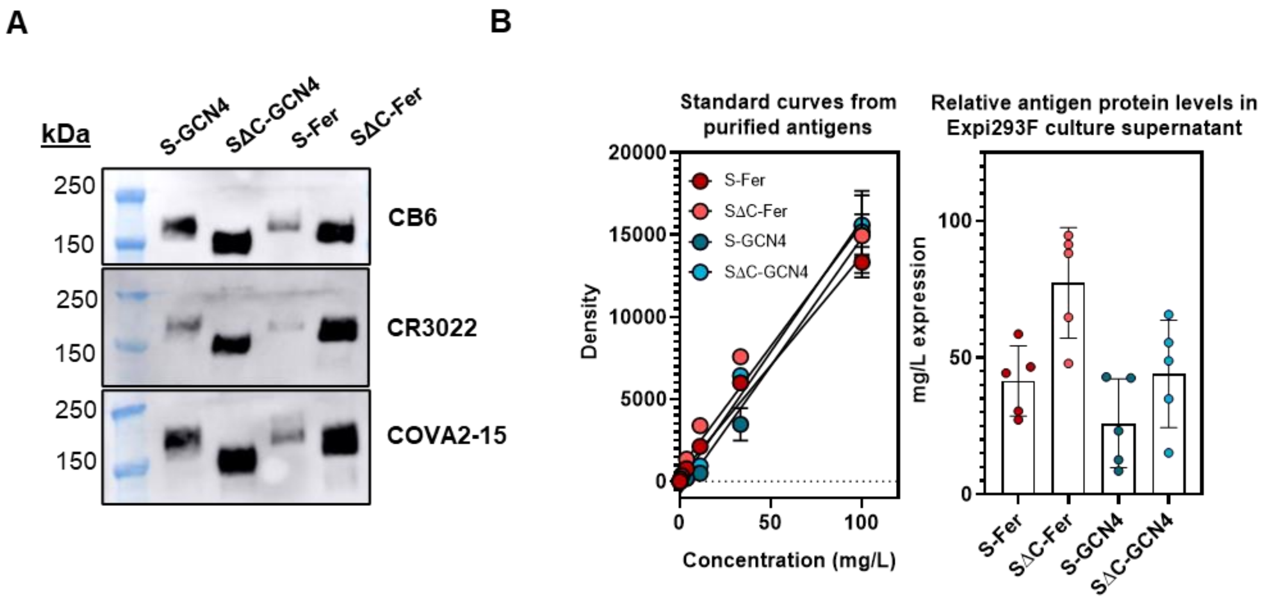

Figure S1. Spike ferritin nanoparticles are expressed at levels similar to those of spike GCN4 trimers. (A) Western blot analysis of Expi293F culture supernatant indicates that expression levels are similar among the spike antigen constructs. Supernatants were blotted with CB6 (top), CR3022 (middle), or COVA2-15 (bottom) SARS-CoV-2 mAbs and read out using an anti-human HRP secondary. (B) Dot blot analysis was performed to estimate protein levels of spike antigens in culture supernatants. Purified antigens were used to generate standard curves using a 3-fold dilution series starting at 0.1 mg/mL (*56*). Dots were quantified using CR3022 primary mAb followed by anti-human HRP secondary. Standard curves were then used to calculate the amount of protein in harvested culture supernatants from 5 replicate protein expressions. The height of the bar is the mean protein concentration from 5 individual protein expression replicates (points) from culture supernatant; error bars represent the standard deviation.

bioRxiv preprint doi: https://doi.org/10.1101/2020.08.28.272518. this version posted August 28, 2020. The copyright holder for this preprint (which was not certified by peer review) is the author/funder. It is made available under a CC-BY 4.0 International license.

A

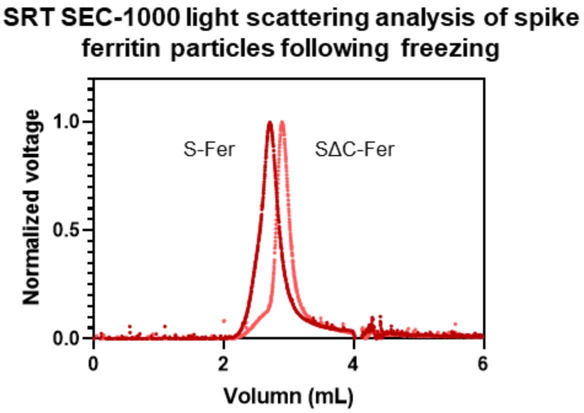

B

Spike ferritin construct	SEC-MALS determined molecular weight (Da)	Predicted molecular weight based on amino acid sequence (Da)
S-Fer	$4.2 \pm 0.17 \times 10^6$	3.7×10^6
SΔC-Fer	$3.1 \pm 0.24 \times 10^6$	3.5×10^6

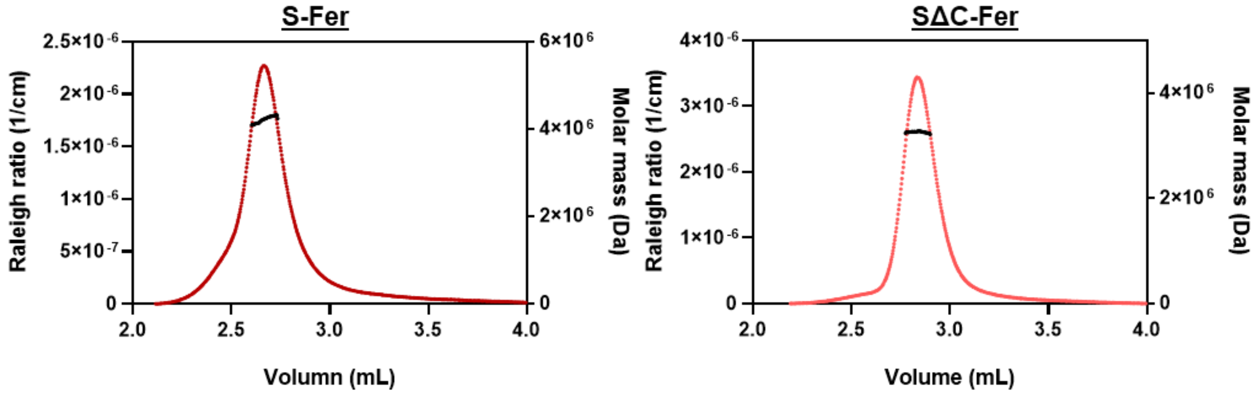

C

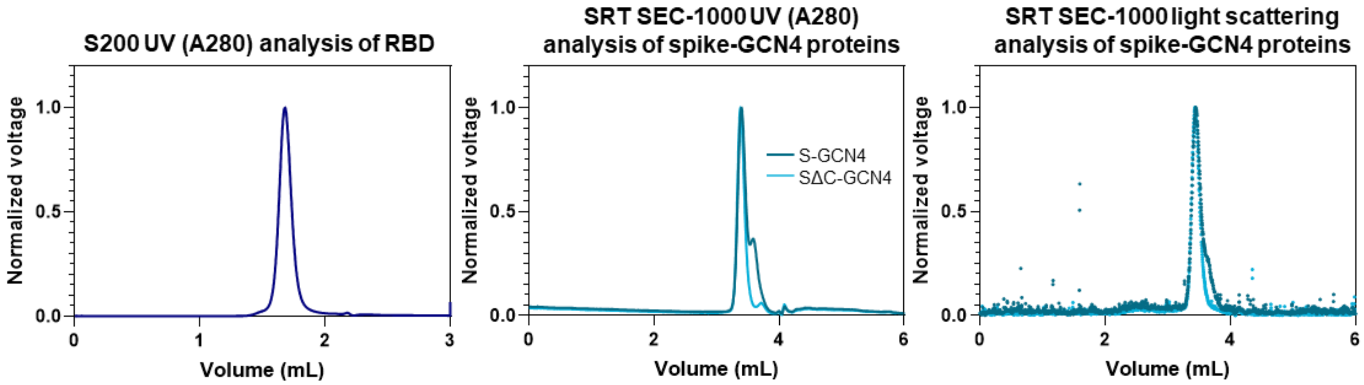

bioRxiv preprint doi: https://doi.org/10.1101/2020.08.28.272518. this version posted August 28, 2020. The copyright holder for this preprint (which was not certified by peer review) is the author/funder. It is made available under a CC-BY 4.0 International license.

Figure S2. Size-exclusion chromatography multi-angle light scattering molecular weight determination of spike ferritin nanoparticles and analysis of other antigens. (A) Light scattering analysis of the S-Fer and SΔC-Fer particles on the SRT SEC-1000 analytical column indicate that particles do not aggregate following a freeze-thaw cycle. Glycerol (10%) was added to particle samples prior to snap-freezing. (B) Molecular weight calculation for S-Fer and SΔC-Fer determined by SEC-MALS was performed with ASTRA software using light scattering and refractive index signals for the particles. The average calculated molecular weight obtained from two independent protein preparations for each particle (S-Fer and SΔC-Fer) is shown. The expected molecular weight was determined by the amino acid sequence for the individual S-Fer and SΔC-Fer protomers and multiplied by 24 to account for the number of protomers in a particle. The expected mass does not account for glycosylation; each protomer contains ~20 predicted N-linked glycans which could add up to 1 MDa to the mass of the particle (*58*). Discrepancies in calculated and expected molecular weights could in part be due to incomplete glycosylation. The plots show a representative curve from each analysis. The colored traces correspond to the left y-axis which shows the Rayleigh ratio, a measure of light scattering. The black line on each peak is the calculated molecular weight, plotted on the right y-axis, as a function of particle elution. This demonstrates that the molecular weight calculation is not subject to variations resulting from artifacts in the eluted peak. (C) SEC-MALS traces for the RBD, S-GCN4, and SΔC-GCN4 demonstrating that samples are pure and do not form aggregates. Only UV A280 is shown for the RBD because it is too small for light scattering to be detected with the miniDAWN detector.

bioRxiv preprint doi: https://doi.org/10.1101/2020.08.28.272518. this version posted August 28, 2020. The copyright holder for this preprint (which was not certified by peer review) is the author/funder. It is made available under a CC-BY 4.0 International license.

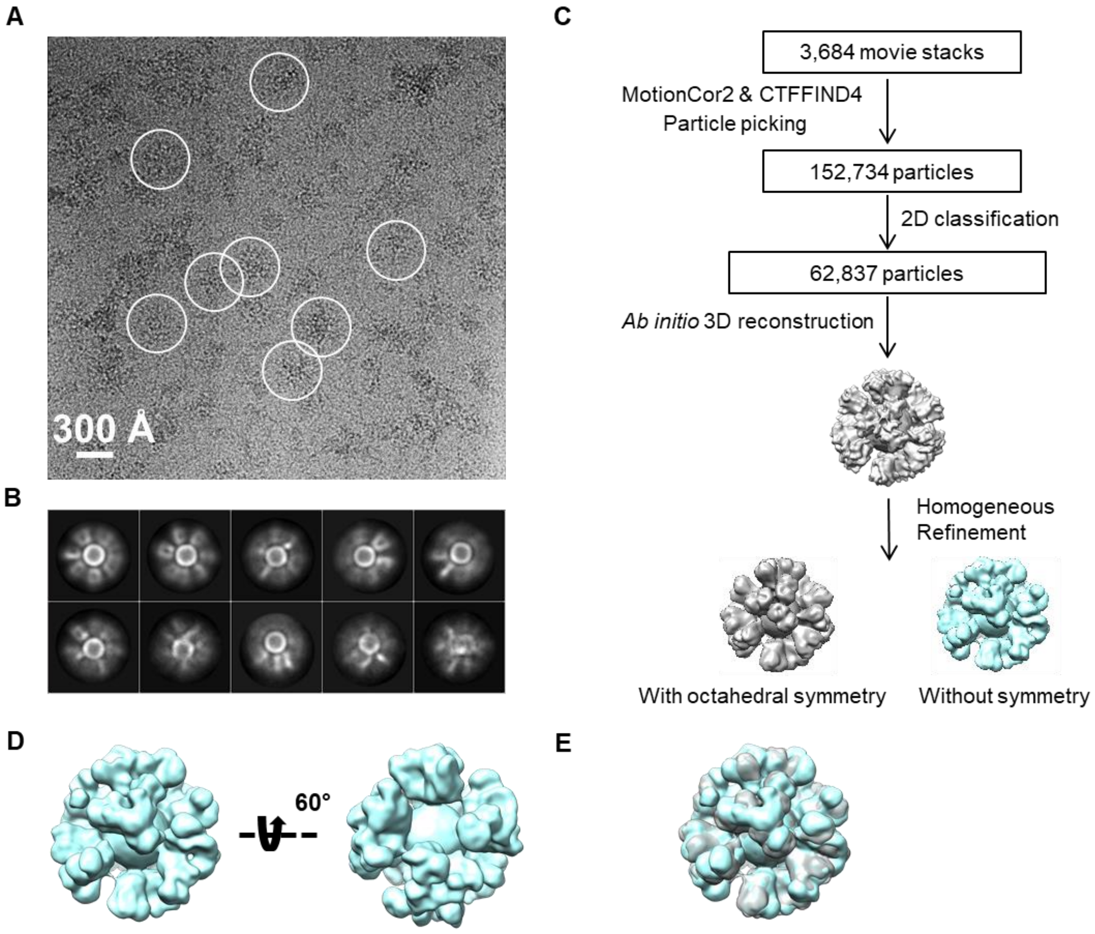

Figure S3. Cryo-EM of S-Fer and SΔC-Fer nanoparticles confirms the presence of spike proteins displayed on the surface of ferritin. (A) Representative motion-corrected cryo-EM micrograph of S-Fer with particles circled in white. (B) Reference-free 2D class averages of S-Fer nanoparticles from analysis of S-Fer indicating the presence of spike on the surface of the particles. (C) Workflow of cryo-EM data processing of SΔC-Fer. (D) Reconstructed cryo-EM map of the SΔC-Fer without symmetry applied (two views). (E) Superimposition (cyan and gray) of the two 3D reconstructions of SΔC-Fer with and without octahedral symmetry demonstrate that the two maps have high similarity, with a cross-correlation coefficient of 0.9857.

bioRxiv preprint doi: https://doi.org/10.1101/2020.08.28.272518. this version posted August 28, 2020. The copyright holder for this preprint (which was not certified by peer review) is the author/funder. It is made available under a CC-BY 4.0 International license.

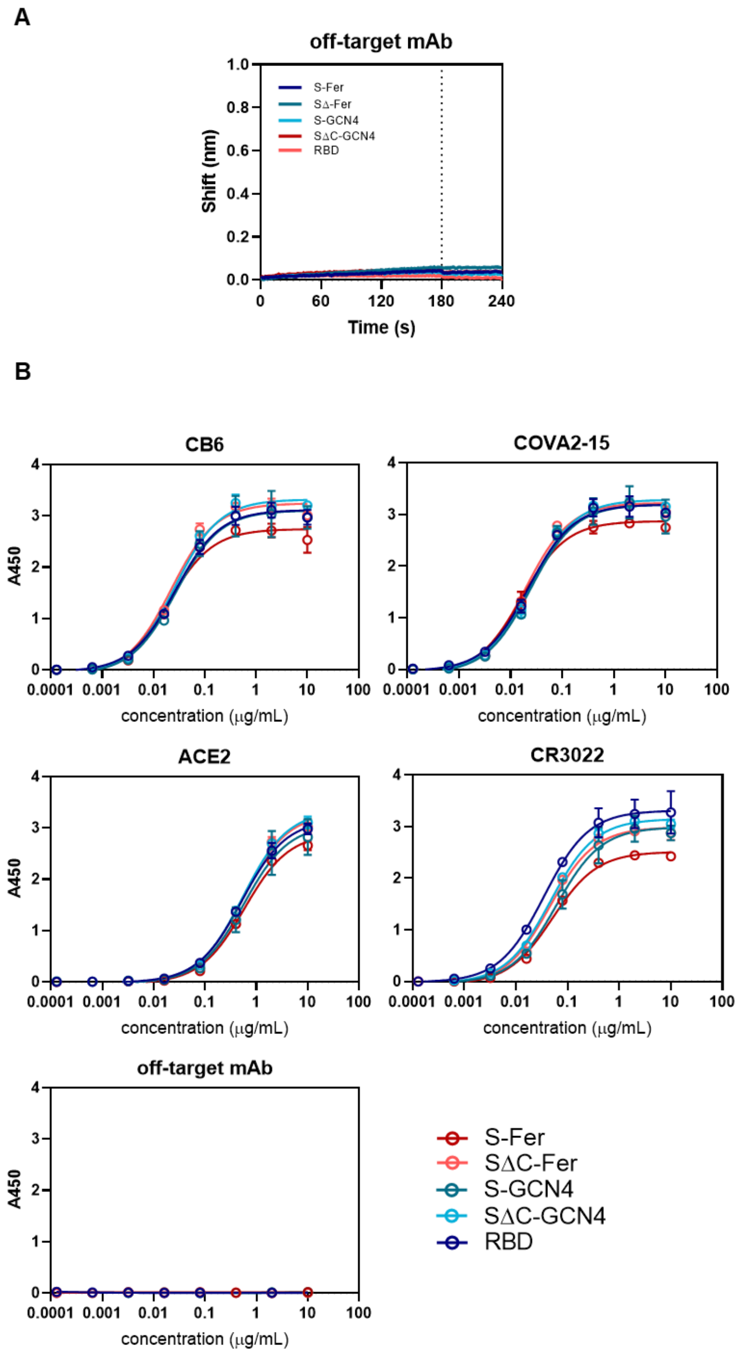

Figure S4. ELISA confirms that the ACE2 binding site and mAb epitopes are displayed on spike ferritin similarly to their display in the RBD and spike trimers. (A) BLI shows that antigens do not bind non-specifically to an off-target Ebola-specific monoclonal antibody, ADI-15731, confirming the specificity of observed binding shown in Figure 3D. (B) For ELISA, antigens were hydrophobically plated at 2 µg/mL and binding of human ACE2 and a set of SARS-CoV-2 antibodies was assessed. ELISA reveals that ACE2 and mAbs bind all antigens in a similar manner. Dilution series of hACE2 and mAbs starting at 10 µg/mL were bound to coated antigens. Binding was quantified using an anti-human-Fc HRP secondary. Each binding curve represents the average binding from 4 replicates and error bars are the standard deviation.

bioRxiv preprint doi: https://doi.org/10.1101/2020.08.28.272518. this version posted August 28, 2020. The copyright holder for this preprint (which was not certified by peer review) is the author/funder. It is made available under a CC-BY 4.0 International license.

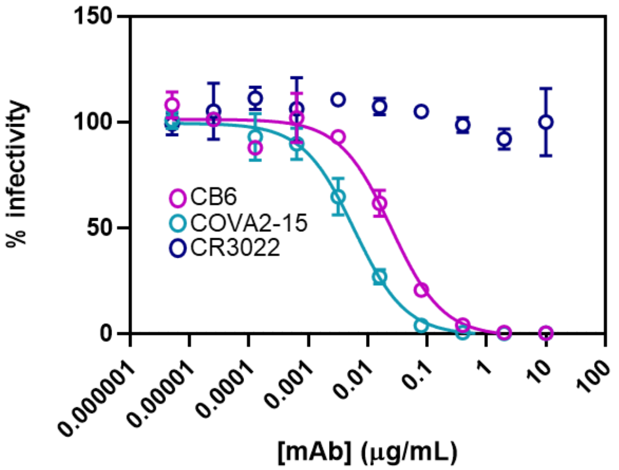

Neutralizing mAb	Determined IC_{50} value (µg/mL)	Literature IC_{50} value (µg/mL)
CB6	0.02 ± 0.009	0.036
COVA2-15	0.004 ± 0.002	0.008

Figure S5. Validation of a SARS-CoV-2 neutralization assay using a spike-pseudotyped lentivirus. The spike pseudotyped lentivirus assay was validated using two published SARS-CoV-2 neutralizing mAbs (CB6 (*55*) and COVA2-15 (*5*)) and one SARS-CoV-2 reactive mAb known to be non-neutralizing (CR3022) (*52*, *53*). CB6 and COVA2-15 dilution curves were fit with a three-parameter non-linear regression to obtain IC_{50} values (Methods). Neutralization assays were performed in technical duplicate or triplicate in 4 independent experiments and one representative curve is shown. Mean IC_{50} values from replicates are shown in the table with standard deviation.

bioRxiv preprint doi: https://doi.org/10.1101/2020.08.28.272518. this version posted August 28, 2020. The copyright holder for this preprint (which was not certified by peer review) is the author/funder. It is made available under a CC-BY 4.0 International license.

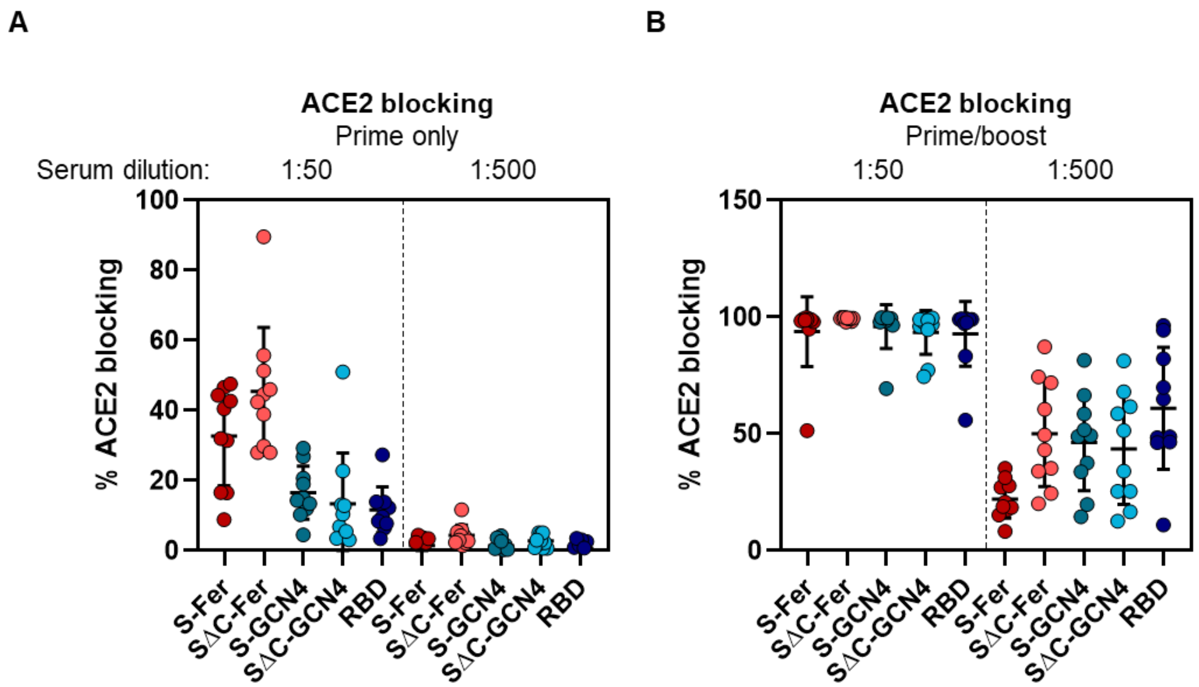

Figure S6. **Sera from mice immunized with SARS-CoV-2 block ACE2 binding to RBD, as indicated by ELISA.** (A) ACE2 blocking activity of sera from mice immunized with a single dose of antigen was determined using an RBD-based ELISA in which RBD-coated plates were incubated with serum dilutions and then ACE2 binding was assayed. ACE2 blocking at a 1:50 and 1:500 serum dilution is shown for each group, and indicates that following a single dose, minimal ACE2 blocking activity is seen in the serum even at a high concentration. No groups show detectable ACE2 blocking activity in the serum diluted at 1:500. Each point represents the average % ACE2 blocking for a single animal assayed in duplicate; each bar represents the mean % ACE2 blocking from the group (*n* = 10 mice per group); error bars represent standard deviation. (B) ACE2 blocking activity was assessed after two doses of antigen and indicates a notable increase in serum antibodies capable of blocking ACE2 binding to RBD. Nearly all ACE2 binding was blocked with a 1:50 serum dilution from all groups, and all groups had detectable blocking at 1:500. Groups and error are as defined in (A).

bioRxiv preprint doi: https://doi.org/10.1101/2020.08.28.272518. this version posted August 28, 2020. The copyright holder for this preprint (which was not certified by peer review) is the author/funder. It is made available under a CC-BY 4.0 International license.

Day 21 RBD ELISA titers

Dunn's multiple comparisons test	Mean rank diff.	Significant?	Summary	Adjusted *P* Value
RBD vs. SΔC-GCN4	3.200	No	*ns*	>0.9999
RBD vs. S-GCN4	-3.300	No	*ns*	>0.9999
RBD vs. SΔC-Fer	-20.70	Yes	*	0.0150
RBD vs. S-Fer	-10.70	No	*ns*	>0.9999
SΔC-GCN4 vs. S-GCN4	-6.500	No	*ns*	>0.9999
SΔC-GCN4 vs. SΔC-Fer	-23.90	Yes	**	0.0025
SΔC-GCN4 vs. S-Fer	-13.90	No	*ns*	0.3299
S-GCN4 vs. SΔC-Fer	-17.40	No	*ns*	0.0761
S-GCN4 vs. S-Fer	-7.400	No	*ns*	>0.9999
SΔC-Fer vs. S-Fer	10.00	No	*ns*	>0.9999

Day 21 Spike ELISA titers

Dunn's multiple comparisons test	Mean rank diff.	Significant?	Summary	Adjusted *P* Value
RBD vs. SΔC-GCN4	-13.50	No	*ns*	0.3837
RBD vs. S-GCN4	-23.20	Yes	**	0.0037
RBD vs. SΔC-Fer	-31.20	Yes	****	<0.0001
RBD vs. S-Fer	-26.10	Yes	***	0.0006
SΔC-GCN4 vs. S-GCN4	-9.700	No	*ns*	>0.9999
SΔC-GCN4 vs. SΔC-Fer	-17.70	No	*ns*	0.0663
SΔC-GCN4 vs. S-Fer	-12.60	No	*ns*	0.5326
S-GCN4 vs. SΔC-Fer	-8.000	No	*ns*	>0.9999
S-GCN4 vs. S-Fer	-2.900	No	*ns*	>0.9999
SΔC-Fer vs. S-Fer	5.100	No	*ns*	>0.9999

Day 28 RBD ELISA titers

Dunn's multiple comparisons test	Mean rank diff.	Significant?	Summary	Adjusted *P* Value
RBD vs. SΔC-GCN4	11.10	No	*ns*	0.8863
RBD vs. S-GCN4	3.200	No	*ns*	>0.9999
RBD vs. SΔC-Fer	0.8000	No	*ns*	>0.9999
RBD vs. S-Fer	20.40	Yes	*	0.0175
SΔC-GCN4 vs. S-GCN4	-7.900	No	*ns*	>0.9999
SΔC-GCN4 vs. SΔC-Fer	-10.30	No	*ns*	>0.9999
SΔC-GCN4 vs. S-Fer	9.300	No	*ns*	>0.9999
S-GCN4 vs. SΔC-Fer	-2.400	No	*ns*	>0.9999
S-GCN4 vs. S-Fer	17.20	No	*ns*	0.0833
SΔC-Fer vs. S-Fer	19.60	Yes	*	0.0264

Day 28 Spike ELISA titers

Dunn's multiple comparisons test	Mean rank diff.	Significant?	Summary	Adjusted *P* Value
RBD vs. SΔC-GCN4	-8.300	No	*ns*	>0.9999
RBD vs. S-GCN4	-22.70	Yes	**	0.0050
RBD vs. SΔC-Fer	-16.60	No	*ns*	0.1089
RBD vs. S-Fer	-4.400	No	*ns*	>0.9999
SΔC-GCN4 vs. S-GCN4	-14.40	No	*ns*	0.2718
SΔC-GCN4 vs. SΔC-Fer	-8.300	No	*ns*	>0.9999
SΔC-GCN4 vs. S-Fer	3.900	No	*ns*	>0.9999
S-GCN4 vs. SΔC-Fer	6.100	No	*ns*	>0.9999
S-GCN4 vs. S-Fer	18.30	Yes	*	0.0500
SΔC-Fer vs. S-Fer	12.20	No	*ns*	0.6129

Table S1. Statistical analysis of spike and RBD ELISA titers from day 21 and day 28 immunization timepoints. Calculated EC_{50} values for each animal for RBD and spike at each time point were compiled by group and assessed using a Kruskal-Wallis ANOVA followed by Dunn's multiple comparisons test. Pairwise comparisons are shown.

bioRxiv preprint doi: https://doi.org/10.1101/2020.08.28.272518. this version posted August 28, 2020. The copyright holder for this preprint (which was not certified by peer review) is the author/funder. It is made available under a CC-BY 4.0 International license.

Day 21 pseudovirus neutralization titers

Dunn's multiple comparisons test	Mean rank diff.	Significant?	Summary	Adjusted P Value
RBD vs. SΔC-GCN4	-1.750	No	ns	>0.9999
RBD vs. S-GCN4	-7.300	No	ns	>0.9999
RBD vs. SΔC-Fer	-26.80	Yes	****	<0.0001
RBD vs. S-Fer	-19.15	Yes	*	0.0122
SΔC-GCN4 vs. S-GCN4	-5.550	No	ns	>0.9999
SΔC-GCN4 vs. SΔC-Fer	-25.05	Yes	***	0.0002
SΔC-GCN4 vs. S-Fer	-17.40	Yes	*	0.0329
S-GCN4 vs. SΔC-Fer	-19.50	Yes	**	0.0099
S-GCN4 vs. S-Fer	-11.85	No	ns	0.4531
SΔC-Fer vs. S-Fer	7.650	No	ns	>0.9999

Day 28 pseudovirus neutralization titers

Dunn's multiple comparisons test	Mean rank diff.	Significant?	Summary	Adjusted P Value
RBD vs. SΔC-GCN4	5.900	No	ns	>0.9999
RBD vs. S-GCN4	2.800	No	ns	>0.9999
RBD vs. SΔC-Fer	-15.70	No	ns	0.1603
RBD vs. S-Fer	-4.000	No	ns	>0.9999
SΔC-GCN4 vs. S-GCN4	-3.100	No	ns	>0.9999
SΔC-GCN4 vs. SΔC-Fer	-21.60	Yes	**	0.0092
SΔC-GCN4 vs. S-Fer	-9.900	No	ns	>0.9999
S-GCN4 vs. SΔC-Fer	-18.50	Yes	*	0.0454
S-GCN4 vs. S-Fer	-6.800	No	ns	>0.9999
SΔC-Fer vs. S-Fer	11.70	No	ns	0.7270

Table S2. Statistical analysis of spike and RBD neuralization titers from day 21 and day 28 immunization timepoints Calculated neutralization IC_{50} values for each animal at each time point were compiled by group and assessed using a Kruskal-Wallis ANOVA followed by Dunn’s multiple comparisons test. Pairwise comparisons are shown.
